# Minute Anatomy of the Kidney

**Published:** 1846-06

**Authors:** 


### PART V.—ABSTRACTS.

### ARTICLE XVI.

### MINUTE ANATOMY OF THE KIDNEY.

By a minute examination of the anatomical structure of the kidneys of one of the higher classes of animals, as of man, we find them composed of a secreting and a vascular apparatus.

The secreting portion, according to Bowman, consists of innumerable convoluted capillary tubes, the tubuli uriniferi. Each of these presents at its origin, where it is connected with the vascular apparatus, a cyst-like dilitation forming a cavity continuous with that of the tube, for the reception of that portion of the vascular apparatus, termed the Malpighian plexus.

The cortical substance of the kidney is composed, principally, of these tubes lined with their secreting cells of epithelium, which, like those of other secreting mucus surfaces, and like the cuticle, are continually being thrown off and renewed. Dr. Johnson has discovered that a very small quantity of fat is deposited in these tubes, even in a healthy condition of the organ.

As remarks Dr. Todd, "the condition of this epithelium affords a most important indication of the state of nutrition of the gland. If its nutrition be perfect, then its secreting functions will be well performed, and the epithelium will be healthy, but if, on the other hand, its nutrition is impaired, one of the results will be unhealthy secretion of epithelium."

The convoluted tubuli uriniferi pass towards the concave surface of the kidney, uniting in their course to form larger and more direct urinary ducts, which converge in groups to several points, thus forming pyramidal shaped bodies, the pyramids of Malpighi. The papillary apex of each pyramid, perforated by numerous openings by which the tubuli discharge their contents, is received into a cup like membranus sack, the calyx. The calices form three groups to communicate with as many cavities of large size, the infundibula, which also communicate with the pelvis.

The vascular apparatus of the kidney is formed from the branches of the renal arteries, which divide and subdivide till they terminate at length in small tubes, the Malphighian arteries. Each of these small vessels passes to one of the dilated extremities of the tubuli uriniferi, pierces through its walls, and, by dividing into numerous very minute capillaries, forms within the cavity a vascular rounded tuft, the Malphigian plexus.

The walls of the minute vessels forming this plexus are thin and transparent, well adapted, evidently, for the passage of fluids. From the interior of the plexus a single vessel takes its origin, which, passing out of the cavity through its walls, breaks up again into numerous small vessels, which form another capillary plexus around the tubuli uriniferi, termed the portal-plexus, so named from the fact that the blood, as in the portal veins, passes through two sets of capillaries and furnishes the material secreted by the kidney, as in the liver, during its passage through the second set of vessels. The following is the language of Dr. Carpenter upon this subject:

"The *cells* lining the Tubuli Uriniferi are probably here, as elsewhere, the instruments by which the *solid* matter of the secretion is elaborated; whilst it can scarcely be doubted that the office of the Corpora Malpighiana is to allow the transudation of the superfluous fluid through the thin-walled and naked capillaries of which they are composed. 'It would, indeed,' Mr. Bowman remarks, 'be difficult to conceive a disposition of parts more calculated to favor the escape of water from the blood than that of the Malpighian body. A large artery breaks up in a very direct manner into a number of minute branches; each of which suddenly opens into an assemblage of vessels of far greater aggregate capacity than itself, and from which there is but one narrow exit. Hence must arise a very abrupt retardation in the velocity of the current of blood. The vessels in which this delay occurs are uncovered by any structure. They lie bare in a cell, from which there is but one outlet, the orifice of the tube. This orifice is encircled by cilia, in active motion, directing a current towards the tube. These exquisite organs must not only serve to carry forward the fluid which is already in the cell, and in which the vascular tuft is bathed, but must tend to remove pressure from the free surface of the vessels, and so to encourage the escape of their more fluid contents.'

"There is a striking analogy between the mode in which the Tubuli Uriniferi are supplied with blood for the purpose of elaborating their secretion, and the plan on which the Hepatic circulation is carried on. The secretion of the Liver is formed from blood conveyed to it by one large vessel, the Vena Portæ which has collected it from the Venous capillaries of the chylopoietic viscera, and which subdivides again to distribute it through the liver. The secretion of the Kidney, in like manner, is elaborated from blood which has already passed through one set of capillary vessels,—those of the Malpighian tufts; this blood is collected and conveyed to the proper *secreting* surface, not by one large trunk (which would have been a very inconvenient arrangement) but by a multitude of small ones,—the *efferent* vessels of the Malpighian bodies, which may be regarded as collectively representing the Vena Portæ, since they convey the blood from the systemic to the secreting capillaries. Hence the Kidney may be said to have a *portal* system within itself."

The renal or emulgent veins are formed of branches from the portal plexus above described.

Some objections were at one time raised to the views of Mr. Bowman, adopted by Dr. Carpenter as quoted as above. Dr. Garlach, however, has more recently thoroughly investigated the subject. The result is that in all of the most important points he has arrived at the same conclusions with Bowman. The only points of difference are noticed in the following quotation from Rankin's Abstract, Part 2d.

"The fact of the direct continuity of the Malpighian capsules with uriniferous tubules, as discovered by Bowman, was clearly confirmed, but it would seem that Bowman's account, which describes the tubules as terminating in these capsules as by so many blind pouches, is not quite correct: the capsules are merely offshoots, or sac-like dilations from the sides of each tubule with the cavity of which they communicate by a slightly narrowed neck, and are not placed at the terminal extremeties of the tubules. Gerlach states that a tubule, having given rise to a pouch, continues its way onwards, forming fresh pouches here and there, and eventually terminates, not in a blind extremity, but by forming a loop; and these loops which the various tubules form have, doubtless, been mistaken for so many blind extremities.

"A curious circumstance remarked by Bowman was that each Malpighian tuft lies quite free within the cavity formed by the capsule, uncovered even by a layer of epithelium: this being the only known instance of a blood-vessel lying bare on a secreting surface, naturally attracted much attention, and was discredited by Reichert and others. Bowman inferred from such an arrangement that the watery and soluble parts of the urine are distilled from the blood of the vessels composing the tufts, whilst the essential elements of the urinary secretion are eliminated by the cells lining the internal surface of the tubuli uriniferi. Gerlach states that he examined this point, and found upon removing the capsule, that the Malpighian tuft was completely covered by a thick layer of nucleated cells. This layer he states to be continued from the one which lines the entire internal surface of the capsule, and which is reflected from this surface on to the Malpighian body, just as the peritoneal coat lining the internal surface of the abdominal cavity is reflected on to the intestines; in both cases a space exists just at the point of reflection which is uncovered by epithelium. The vessels composing the Malpighian tuft therefore are in immediate contact with, or imbedded in a thick layer of nucleated cells, which, doubtless, are actively engaged in carrying on the process of urinary secretion."

